# New Insights into Artesunate as a Pleiotropic Regulator of Innate and Adaptive Immune Cells

**DOI:** 10.1155/2022/9591544

**Published:** 2022-02-07

**Authors:** Lixian Lin, Zengqi Tang, Zhenrui Shi, Qing Guo, Hui Xiong

**Affiliations:** Department of Dermatology, Sun Yat-sen Memorial Hospital, Sun Yat-sen University, Guangzhou, Guangdong 510120, China

## Abstract

Artesunate, one of the derivatives of artemisinin (“qinghaosu” in Chinese), is known as an antimalarial drug with high efficiency and low toxicity. Of interest, emerging evidences suggest that artesunate also possesses an immunomodulatory effect during innate and adaptive immune responses in cell types and context-dependent manner. Although it shows promising application in many diseases, such as inflammatory diseases, hypersensitivity, autoimmune diseases, and cancers, little is known about underlying molecular. In this review, we summarize recent advances of how artesunate regulates innate and adaptive immune cells. In addition, its potential application in immune-related diseases is also highlighted.

## 1. Introduction

Artesunate is a semisynthetic derivative of artemisinin (“qinghaosu” in Chinese), the active component of *Artemisia annua*. Due to its rapid onset, high efficiency, and low toxicity, artesunate was approved as a new antimalarial drug by the Chinese Ministry of Health in 1987 [1]. Of note, artesunate was reported to be effective for the treatment of cerebral malaria [2]. Autoimmunity is a critical pathogenic process of cerebral malaria. Thus, artesunate may also display immunomodulatory function in addition to the clearance of malaria parasites, which has been exclusively studied for decades. In this paper, we review the studies of functions of artesunate in specific immune cells including its potential application in treating immune-pathology diseases (Tables 1 and 2).

## 2. Artesunate and Innate Immune Cells

### 2.1. Artesunate and Neutrophils

As the most abundant leukocytes in the blood, neutrophils play a key role in innate immunity. Neutrophils are involved in multiple biological processes during acute tissue damage and infection, such as chemotaxis, phagocytosis, and sterilization.

Researchers found that neutrophil counts were decreased in patients who took artesunate during antimalarial clinical trials [3–6]. Similar results were also observed in experimental animal models. Artesunate reduced the infiltration of neutrophils and ameliorated the inflammatory symptoms in the monoarthritis rat model [7], ovalbumin-induced allergic asthma model [8], and septic lung injury mouse model [9]. Mechanistically, artesunate promoted the expression of nuclear factor E2-related factor 2 (Nrf2); reduced the expression of TNF-*α*, IL-6, COX-2, iNOS, and NADPH oxidases (NOX1, NOX2, NOX3, and NOX4); and suppressed the oxidation of regulatory subunits p22phox and p67phox of NOX Pro. In vitro, it suppressed the chemotaxis of neutrophils in human peripheral blood which was induced by IL-8 [10]. In brief, artesunate can reduce the infiltration and oxidative damage of pathogenic neutrophils, resulting in a nonspecific anti-inflammatory effect.

However, some studies revealed inconsistent conclusions. In vitro, artesunate promoted the reactive oxygen species (ROS) response of neutrophils with *Escherichia coli* infection [11]. The neutrophil counts were significantly increased in the blood of mice exposed to low-dose artesunate (8 mg/kg/d) for 45 days [12], suggesting the effect of artesunate on neutrophils is in an immune state-, dosage-, and time-dependent manner, whose specific mechanism remains to be investigated in the future.

### 2.2. Artesunate and Monocytes/Macrophages

Monocytes circulating in the blood and macrophages in tissues have strong phagocytic capacity. Monocytes/macrophages also act as antigen-presenting cells, which play a key role in immune response. The activation of Toll-like receptor (TLR) and lipopolysaccharide (LPS) receptors on the surface of monocytes/macrophages leads to various proinflammatory cytokines such as TNF-*α*, NO, IL-1*β*, IL-6, and IL-12 which are responsible for mediation by the NF-*κ*B signaling pathway [54, 55].

It is well studied that artesunate interrupted the NF-*κ*B transcriptional signaling pathway, resulting in reduced synthesis and secretion of proinflammatory cytokines in monocytes/macrophages. Indeed, artesunate inhibited TNF-*α* and IL-6 production in a dose-dependent manner in peritoneal macrophages of the sepsis mouse model via inhibiting the expression of TLR2 and nucleotide-binding oligomerization domain 2 (Nod2) and suppressing NF-*κ*B activation [13, 14]. Similarly, in the experimental colitis model, inflammatory cytokines such as IL-17, IFN-*γ*, and TNF-*α* levels were significantly decreased in monocytes/macrophages in mice treated with artesunate, presumably due to the downregulation of NF-*κ*B p65 and p-I*κ*B-*α* levels [15]. In the human monocytic cell line (THP-1 cells), artesunate could alleviate the inflammation state through the suppression of NF-*κ*B transcription and downregulation of metalloproteinase- (MMP-) 9 expression [17].

Artesunate can also attenuate the proinflammatory effect of monocytes/macrophages via noncanonical pathways. In macrophage RAW 264.7 mouse cell lines, artesunate considerably inhibited NO production by promoting the cAMP-mediated signaling pathway while suppressing the Wnt/*β*-catenin signaling pathway [18]. Moreover, artesunate inhibited autophagic activation of monocytes/macrophages via the tumor necrosis factor receptor-associated factor 6- (TRAF6-) Beclinl-phosphatidylinositol 3-kinase class III (PI3KC3) pathway. More specifically, artesunate inhibited K63-linked ubiquitination of Beclin1 and subsequently disrupted the complex of Beclin1–PI3KC3 [19]. In addition, artesunate enhanced scavenger receptor (SR) mRNA expression and LPS internalization in macrophages, which reduced proinflammatory cytokines and serum LPS levels in cecal ligation/puncture sepsis mouse model [16].

However, artesunate can reverse the immunosuppression of macrophages in sepsis. Sepsis undergoes a transit from an early inflammatory state to a persistent immunosuppressive state, which is characterized by LPS tolerance. With LPS tolerance, the autophagy activity of macrophages declines sharply and cytokines production decreases, which results in impairment of bacterial clearance. Artesunate could restore Unc-51-like autophagy-activated kinase 1- (ULK1-) mediated autophagy, reverse LPS tolerance, and relieve the immunosuppression of macrophages induced by sepsis. The interference of the CaMKII-IP3R-CaMKK*β* pathway [20] and the activation of vitamin D receptor [21] were found involved in the process.

Thus, artesunate may have a two-way immunomodulatory effect on monocytes/macrophages. On the one hand, artesunate affects the gene expression and proinflammatory cytokine production of monocytes/macrophages via various signaling pathways, which has a certain protection in the occurrence of tissue inflammatory injury; on the other hand, artesunate can restore the reactivity of macrophages, which has a potential therapeutic effect on immunosuppression caused by sepsis.

### 2.3. Artesunate and NK Cells

Natural killer (NK) cells can eliminate virus-infected cells and tumor cells nonspecifically, which play an important role in immune defense and surveillance.

Artesunate significantly enhanced the cytotoxic activity of NK cells against tumor cells via suppressing the expression of transforming growth factor-*β*1 (TGF-*β*1) and IL-10 [22–26]. Thus, artesunate may be a promising approach for antitumor therapy to reverse the immunosuppression activity of cancer cells.

### 2.4. Artesunate and Mast Cells

Mast cells are mainly distributed around the submucocutaneous microvessels and the capsule of visceral organs. The high-affinity IgE Fc receptors (Fc*ε*R I) on their surface mediate type I hypersensitivity.

In vitro, artesunate inhibited IgE-mediated degranulation of RBL-2H3 mast cell lines and mature human mast cells in a dose-dependent manner. In addition, artesunate inhibited IgE-induced tyrosine kinase Syk and PLC*γ*1 phosphorylation, IP3 production, and cytoplasmic Ca^2+^ levels in the ovalbumin-induced bronchial asthma guinea pig model [27]. These findings suggested that artesunate can be applied in treating allergic asthma and other mast cell-mediated anaphylactic diseases.

### 2.5. Artesunate and *γδ*T Cells

*γδ*T cells are special T cells whose T cell receptor (TCR) is composed of *γ* and *δ* chains. They participate in innate immune response with cytotoxic effect and antigen-presenting function. In addition, activated *γδ*T cells produce a variety of cytokines to enhance immune response.

Artesunate reduced the *γδ*T cell population in the draining lymph nodes and consequently alleviated systemic inflammation in the imiquimod-induced psoriasis-like dermatitis mouse model [28].

Nonetheless, artesunate can reverse the immunosuppressive effect of tumor cells on *γδ*T cells. It increased the expression of granzyme B (GZMB) in *γδ*T cells and reduced TGF-*β*1 levels as well as upregulated the expression of Fas in HepG2 cells. Consequently, the killing effect of *γδ*T cells on tumor cells were reinforced [29]. Taken together, the immunomodulation effect of artesunate on *γδ*T cells is in a cell type- and microenvironment-dependent manner.

### 2.6. Artesunate and Eosinophils

The number of eosinophils in the mucosa is about 100 times of that in peripheral blood. They contain eosinophilic granules with various kinds of enzymes and express complement receptors and low-affinity Fc*ε*RII on the surface. They can resist type I hypersensitivity and infection of parasites and virus as well as generate inflammation.

In the female BALB/c mouse model of allergic asthma sensitized by ovalbumin and activated by aerosol inhalation, artesunate dose dependently reduced eosinophil counts and their productions (IL-4, IL-5, IL-13, and eotaxin) [30]. More specifically, it blocked the epidermal growth factor- (EGF-) induced phosphorylation of Akt and its downstream substrates-tuberin, p70S6 kinase, and 4E-binding protein 1, thus inhibiting the activation of NF-*κ*B and the release of proinflammatory cytokines [31]. Another study found that artesunate promoted the apoptosis of eosinophils in the lung tissue via increasing the level of Fas and suppressing the expression of Bcl-2 [32]. Therefore, artesunate can reduce the number and proinflammatory cytokine secretion of eosinophil as well as promote eosinophil apoptosis, which provided the potential of artesunate in the treatment of allergic asthma.

## 3. Artesunate and Adaptive Immune Cells

### 3.1. Artesunate and T Cells

T cell-mediated immune response can be divided into three stages: (1) specific recognition of antigens and (2) activation, proliferation, and differentiation. Double signals activate T cells, which stimulate the synthesis of IL-2 and CD25 (*α* chain of IL-2 receptor). The interaction between IL-2 and IL-2 receptors through autocrine or paracrine is the key to initiate and promote the proliferation of T cells [56]. The nature of antigen and the type of cytokines secreted determine the subsets of T cell differentiation. (3) Effector T cells work. T cells can be divided into three subsets according to their functions. ① Auxiliary function: T helper cells (Th) secrete diverse cytokines to assist other immune cells to work. For example, Th1 cells assist cellular immunity while Th2 cells assist humoral immunity. ② Killing function: cytotoxic T cells (CTL) can kill target cells directly and specifically. ③ Inhibitory function: regulatory T cells (Treg) suppress immune function [57]. Once pathogens are cleared, proinflammatory cytokines stop releasing, activated T cells start apoptosis, and consequently, the host returns to immune homeostasis.

More and more evidence support the immunosuppression of artesunate on T cells. First, artesunate regulates activation and proliferation of T cells. In vitro, artesunate reduced the percentage of activated CD4^+^ and CD8^+^ T cells after PHA stimulation [35]. Its inhibition of T cell activation and proliferation could also be observed in the experimental autoimmune myasthenia gravis rat model [33] and delayed type hypersensitivity (DTH) mouse model [36, 37]. More specifically, artesunate reduced costimulatory molecule CD86 expression [33], restrained the production of IL-2, CD25 (IL-2 receptor *α* chain) and CD69 on CD4^+^ T cells, and then suppressed the activation of naïve T cells [34].

Second, artesunate modulates differentiation of T cells. Th1/Th2 and Treg balance was involved in the cerebral malaria mouse model [38, 39], the experimental autoimmune myasthenia gravis rat model [33], and the nonobese diabetic mouse model of type 1 diabetes mellitus (T1DM) [40] treated with artesunate. Artesunate increased the number of Treg cells and their production of IL-10 and TGF-*β*, which might be related to the enhanced activation of NF-*κ*B p65 and Smad2/3 [36, 37]. Meanwhile, the number and the immune response of Th1 cells were reduced in target organs, accompanied by downregulation of proinflammatory cytokines such as TNF-*α* and IL-6 [40]. Moreover, Th17/Treg balance was involved in the rheumatoid arthritis rat model [41], the chronic graft-versus-host disease mouse model [42], and the atopic dermatitis mouse model [43] treated with artesunate. Artesunate increased the number of Treg cells, induced Th17 cells apoptosis, and restrained the immune response of Th17 cells. Mechanistically, artesunate promoted the expression of SOCS3 protein and inhibited the phosphorylation of ROR*γ*t and STAT3 in Th17 cells. Subsequently, Th17 cells synthesized less proinflammatory cytokines such as IL-6 and IL-17 [43]. In addition, artesunate suppressed the differentiation of spleen follicular helper T cells (Tfh cells) and the activation of JAK2-STAT3 signaling pathway in the lupus-prone MRL/lpr mice [44].

Third, artesunate inhibited the pathogenic T cell migration to the brain and then attenuated the infiltration of inflammatory cells in the cerebral malaria mouse model [38, 39] and the experimental autoimmune encephalomyelitis mouse model of multiple sclerosis [45]. The immunosuppressive effect of artesunate on T cells suggests its study value in treating T cell-mediated diseases.

In contrast, artesunate can reinforce immune response of T cells in immunosuppressive states. In vitro, it promoted the production of IFN-*γ* or IL-4 in differentiated Th1 or Th2 cells, respectively, which enhanced the effector T cells response [34]. The promotion of Th1 differentiation from CD4^+^ T cells was also suggested in the ovarian cancer mouse model treated with artesunate. Mechanistically, artesunate upregulated miR-142 expression and reduced Sirt1 levels in peripheral CD4^+^ T cells [46]. In addition, it reduced PGE2 production and Foxp3 expression in T cells and subsequently decreased the percentage of Treg cells, leading to enhanced antitumor effect of immune system in the cervical cancer mouse model [47] and the ret-transgenic mouse model of melanoma [48]. Moreover, artesunate could significantly accelerate immune reconstitution in the mice after bone marrow transplantation with long-term deficiency of T cell function [49]. Of note, as an antimalarial drug, artesunate could not only kill the parasite directly but also induced a significant increase in the number of CD8^+^ effector memory T cells (Tem cells) in the liver, which protected the *Plasmodium yoelii*-infected mice against the second challenge of sporozoites [50].

At present, drugs for autoimmune diseases mainly produce an immunosuppressive effect, but we cannot ignore their side effects to increase the risk of opportunistic infection and tumors [58]. For its two-way immunomodulation on T cells, artesunate may help reinstate immune homeostasis without the above risks. Therefore, artesunate has some advantages in the treatment of T cell-mediated autoimmune diseases, and can also be applied in tumor immunotherapy and immune reconstruction of T cell immune deficiency. However, the mechanisms of the two-way immunomodulation of artesunate on T cells are unclear, which need more evidence to elucidate.

### 3.2. Artesunate and B Cells

B cells mediate humoral immunity by synthesizing and secreting specific antibodies. However, the production of autoantibodies in some pathological conditions results in autoimmune diseases.

Artesunate can inhibit pathological B cells generating autoantibody. In the MRL/lpr mouse model of SLE, artesunate reduced the serum levels of antinuclear antibody (ANA) and anti-dsDNA antibody titer by inhibiting B cell activating factor (BAFF) expression in the spleen [51]. Further, it was found that artesunate targeted the germinal center (GC), where the autoantibody is produced in a K/BxN mouse model of rheumatoid arthritis. In this way, the number of B cells in GC decreased significantly accompanied with descending level of serum autoantibody [52]. Moreover, artesunate decreased IL-6 levels and increased TGF-*β* levels to almost normal in the serum, which reversed the overactive B cells to generate autoantibody stimulated by high level IL-6, and restored the inhibitory effect of TGF-*β* on lymphocyte proliferation [53]. These results provide a new proof for artesunate to treat autoantibody-mediated autoimmune diseases.

## 4. Summary and Prospect

Artesunate has an immunomodulatory effect on various immune cells and cytokines of immune system, but it shows different regulatory effects in different immune states. For example, artesunate inhibits the overactive immune response in autoimmune diseases, while it enhances the killing effect of the immune system on tumor cells. Few studies involved the mechanism of this difference. The immunomodulation of artesunate affects diverse molecules of signaling pathways, including TLR, PLC*γ*, Akt, Wnt, STATs, NF-*κ*B, and Nrf2. However, the direct target molecules and molecular mechanisms of artesunate are still unclear, which needs further exploration. In addition, artesunate has the advantages of low toxicity and price. At present, many in vitro and animal studies support it as a promising candidate to treat inflammatory diseases, hypersensitivity, autoimmune diseases, and cancers. However, more evidence is needed in further clinical trials to evaluate the efficacy and safety of artesunate.

## Figures and Tables

**Table 1:** Effects of artesunate on some innate immune cells and potential application.

Cell type	Observed effects	Signal pathways	Potential application
Neutrophils	Reduction of counts [3–7] Inhibition of oxidative response [8, 9] Reduction of proinflammatory cytokines [9] Inhibition of chemotaxis [10] Promotion of oxidative response with infection [11] Promotion of counts with low dose for long time [12]	Nrf2 signaling pathway [8]	Monoarthritis [7] Allergic asthma [8] Septic lung injury [9]

Monocytes/macrophages	Reduction of proinflammatory cytokines [13–16] Reduction of MMP-9 [17] Reduction of NO [18] Inhibition of autophagic activation [19] Promotion of LPS internalization [16] Restoration of autophagy and reversing LPS tolerance in sepsis [20, 21] Activation of vitamin D receptor [21]	NF-*κ*B pathway [13–15, 17] cAMP-mediated pathway [18] Wnt/*β*-catenin pathway [18] TRAF6-Beclinl-PI3KC3 pathway [19] CaMKII-IP3R-CaMKK*β* pathway [20]	Sepsis [13, 14, 16, 17, 20, 21] Colitis [15]

NK cells	Promotion of cytotoxic activity against tumor cells [22–26] Reduction of TGF-*β*1 and IL-10 [22]		Tumor [22–26]

Mast cells	Inhibition of IgE-mediated degranulation [27]	IP3/Ca^2+^ signaling pathway [27]	Allergic asthma [27]

*γδ*T cells	Reduction of population in draining lymph nodes [28] Promotion of cytotoxic activity on tumor cells [29] Reduction of TGF-*β*1 [29]		Psoriasis [28] Tumor [29]

Eosinophils	Reduction of counts [30] Reduction of IL-4, IL-5, IL-13, and eotaxin [30, 31] Promotion of apoptosis [32]	PI3K/Akt pathway [31] NF-*κ*B signaling pathway [31]	Allergic asthma [30–32]

**Table 2:** Effects of artesunate on adaptive immune cells and potential application.

Cell type	Observed effects	Signal pathways	Potential application
T cells	Inhibition of activation of naïve T cells [33, 34] Inhibition of proliferation [33–37] Promotion of Treg differentiation and production of IL-10 and TGF-*β* [36–42] Reduction of Th1 differentiation and their immune response [40] Reduction of proinflammatory cytokines [40, 43] Promotion of Th17 apoptosis and inhibition of their immune response [41–43] Reduction of Tfh differentiation [44] Inhibition of pathogenic T cell migration [38, 39, 45] Promotion of effector Th cell response [34] Promotion of antitumor effect [46–48] Promotion of T cell function reconstitution [49] Promotion of Tem cells against malaria [50]	NF-*κ*B signaling pathway [36, 37] Smad2/3 signaling pathway [36, 37] JAK2/STAT3 pathway [44]	Myasthenia gravis [33] Delayed type hypersensitivity [36, 37] Cerebral malaria [38, 39] Type 1 diabetes mellitus [40] Rheumatoid arthritis [41] Chronic graft-versus-host disease [42] Atopic dermatitis [43] Lupus nephritis [44] Autoimmune encephalomyelitis [45] Tumor [46–48] Immune function recovery [49]

B cells	Inhibition of BAFF expression and autoantibody generation [51–53] Promotion of TGF-*β* [52] Reduction of IL-6 [52] Reduction of B cells in germinal center [53]		Systemic lupus erythematosus [51] Rheumatoid arthritis [53]
